# Investigation of Potential Cross-Protection Conferred by the Seasonal Influenza Vaccine Against Swine Influenza A Viruses of Pandemic Potential

**DOI:** 10.3390/vaccines14030211

**Published:** 2026-02-26

**Authors:** Alice Lilley, Chiara Chiapponi, Alice Prosperi, Ana Moreno, Laura Soliani, Nicola Lewis, Ruth Harvey

**Affiliations:** 1 The Worldwide Influenza Centre, The Francis Crick Institute, London NW1 1AT, UK; nicola.lewis@crick.ac.uk (N.L.); ruth.harvey@crick.ac.uk (R.H.); 2 WOAH Reference Laboratory for Swine in Influenza, Istituto Zooprofilattico Sperimentale Della Lombardia e dell’Emilia Romagna, 25124 Brescia, Italy; chiara.chiapponi@izsler.it (C.C.); alice.prosperi@izsler.it (A.P.); anamaria.morenomartin@izsler.it (A.M.); laura.soliani@izsler.it (L.S.)

**Keywords:** swine influenza A virus, seasonal influenza vaccine, cross-reactive antibodies, pandemic preparedness

## Abstract

**Background/Objectives**: Influenza A viruses cause seasonal epidemics of respiratory infections in humans, the severity of which can be mitigated by influenza vaccine use. Influenza A viruses circulating in pigs continue to pose a pandemic threat, as evidenced by the influenza virus that caused the 2009 pandemic, which originated in pigs. To understand the relative risk of emergence of influenza A viruses from pigs and to assess the potential role of the seasonal influenza vaccine in mitigating this risk, we evaluate the potential cross-protection afforded by the seasonal influenza vaccine against different clades of recently circulating swine influenza A viruses. **Methods**: The presence of cross-reactive antibodies in pre- and post-vaccination human serum samples was measured in haemagglutination and microneutralisation assays. Representative H1 swine influenza A viruses from different genetic lineages were tested against sera collected after administration of the seasonal influenza vaccine in healthy adult volunteers over a 6-year time-period. **Results**: Although a clade-dependent boosting of post-vaccination antibody titres was observed, protective titres often failed to be reached. There was heterogeneity in recognition by sera for the contemporary swine influenza A viruses, with the 1C.2.1 clade virus being well recognised in both assays, whilst very low pre- and post-vaccination antibody titres were observed against the 1A.3.3.2 clade (which emerged in pigs following the reverse zoonotic introduction from humans of the A/H1N1 pdm09 virus) by both assays. **Conclusions**: Seasonal influenza vaccines produce cross-reactive antibodies against some clades of influenza A viruses circulating in pigs, but not all. Depending on the lineage and clade of the virus, the seasonal influenza vaccine might have utility in the event of a swine variant outbreak in humans, whilst a specific vaccine against the outbreak strain is developed.

## 1. Introduction

Influenza A viruses cause seasonal epidemics globally, with variable morbidity and mortality [1,2]. Vaccination is the most effective method of reducing influenza-related morbidity and mortality. Influenza A viruses (IAV) are found in a wide range of animal reservoirs, including birds and pigs [3]. Swine influenza A viruses (SwIAV) affect swine populations globally, causing morbidity in pig populations and presenting a risk of transmission to humans [4,5]. The last influenza pandemic in 2009 was caused by a SwIAV of the H1N1 subtype (H1N1pdm09) [6]. It has been estimated that over 280,000 deaths were caused by respiratory and cardiovascular complications associated with the 2009 SwIAV pandemic in the first 12 months alone [7].

SwIAV evolves more slowly than human IAV, and in a unique manner that shows greater geographic variation, even between farms in a given location [8]. A complex and dynamic relationship exists between human seasonal IAV and SwIAV; surveillance of swine populations demonstrates the periodic spill-over and onward transmission of human seasonal IAV into swine populations, expanding its genetic diversity in pigs [5,9]. Frequent outbreaks of SwIAV occur in swine populations as well as occasional detections of swine-origin influenza A virus infections in humans—termed variant infections [10,11]. Swine-origin influenza A viruses infecting humans are termed “variant” and are distinguished from human seasonal subtypes by the letter “v” following the subtype or strain name [12]. Due to a lack of diagnosis and asymptomatic cases, there is likely under-reporting of human variant infections [13].

In Europe, sporadic variant infections have been reported. In the UK, a variant infection caused by a strain belonging to the 1B.1.1 lineage (H1N2) was detected in November 2023 [14]. Human cases of swine-origin influenza A viruses of the 1C (Eurasian avian-like) lineage have been detected in several European countries, including Italy, the Netherlands, Germany, and Switzerland [15,16,17,18,19]. Additionally, a human case of swine-origin influenza A virus infection, caused by a virus of the 1A.3.3.2 (H1pdm09-like) clade, was detected in Denmark in 2021 [20]. These sporadic human cases highlight the ability of SwIAV to infect humans, which, in combination with the risk of onward transmission to cause an epidemic or even a pandemic, justifies the need for SwIAV pandemic preparedness.

Assessments of the potential cross-protection and/or cross-reactivity afforded by seasonal influenza vaccines have been undertaken previously. Van Diemen et al. [21] found that ferrets vaccinated with the 2016–2017 seasonal influenza vaccine were protected from infection with an H1N1 SwIAV of the 1A (classical) lineage. However, ferrets who received the same vaccination were not protected from infection with a 1C lineage H1N1 SwIAV. Post-vaccination human sera collected between 2005 and 2009 did not contain cross-reactive antibodies against an H1N1pdm09 virus when tested in a virus microneutralisation assay [22]. Henritzi et al. [23] tested SwIAV isolated from domestic pig populations in Europe against 2018/2019 season post-vaccination human sera in microneutralisation assays. Results showed high cross-reactive antibody titres against SwIAV from the 1A, 1B (human-like) and 1C lineage, with titres matching those seen against the vaccine strain. However, decreased titres were observed against one 1C lineage SwIAV. Only post-vaccination serum samples were tested, and therefore, the effect of vaccination on microneutralisation titres could not be assessed. The established correlate of protection (CoP) is a haemagglutination inhibition assay (HI) titre of ≥ 1:40, and a 4-fold rise in HI titre is commonly used as a marker for seroconversion [24,25]. However, previous studies have not included HI data.

The lack of human serological data assessing the potentially immunological recognition afforded by the seasonal influenza vaccine against currently circulating H1 SwIAV leaves a gap in pandemic preparedness. Although SwIAV candidate vaccine viruses are regularly reviewed to determine their suitability as vaccine strains, seasonal influenza vaccines are readily available and quicker to produce due to existing manufacturing pipelines [26]. Here, the ability of the seasonal influenza vaccine to generate cross-reactive antibodies against contemporary European H1 SwIAV was measured using both haemagglutination inhibition (HI) and microneutralisation (MN) assays to understand the utility of the seasonal influenza vaccine in the event of a SwIAV outbreak. The value of an emergency seasonal influenza vaccine booster in the case of an outbreak was assessed by the use of longitudinal human sera panels to observe whether antibody titres wane between vaccinations.

## 2. Materials and Methods

### 2.1. Human Sera Samples

Pre- and post-vaccination human sera samples were collected over six consecutive years, pre-vaccination and two to four weeks post-vaccination, from anonymised volunteers working at the Francis Crick Institute, London. No details of age, previous vaccination or infection history, or exposure to swine were collected, and no samples were excluded from this study. Samples were collected in BD Vacutainer SSTII tubes. Blood samples were spun for 10 min at 2000 rpm before serum supernatants were removed and heat-inactivated at 56 °C for 30 min. All serum samples were treated with receptor-destroying enzyme (RDE [Denka-Seiken, Tokyo, Japan]) to remove non-specific inhibition and stored at −20 °C until use. Use of these sera was approved by The Francis Crick Institute Internal Ethics Review Committee, registration number 2019FC5. Serum panel sizes by year can be seen in Supplemental Table S1.

### 2.2. Virus Propagation

Swine viruses were selected as representatives of different swine H1 clades and lineages. All viruses were held at the Worldwide Influenza Centre at the Francis Crick Institute, London, UK. Swine viruses were collected and shared by colleagues at Istituto Zooprofilattico Sperimentale della Lombardia e dell’Emilia Romagna, Italy. Confluent MDCK-SIAT1 [27] monolayers in T75 flasks were washed twice with DMEM containing 5% penicillin and streptomycin (DMEM + PS) before inoculation with 2 mL of virus diluted to 10^−3^ in DMEM + PS and incubated for 30 min at room temperature. A total of 50 mL of DMEM + PS containing 2 µg/mL of tosyl phenylalanyl chloromethyl ketone (TPCK)-treated trypsin was added to each flask and incubated for three days at 37 °C with 5% CO_2_. Sequencing of virus stocks was carried out prior to use in assays.

The viruses used are included in Table 1. Vaccine strain viruses were held at the Worldwide Influenza Centre at the Francis Crick Institute, London. Egg- or cell-grown strains were selected based on the type of vaccine used at the Francis Crick Institute in the respective years.

### 2.3. Haemagglutination Inhibition (HI) Assay

HI assays were carried out in accordance with World Health Organisation standardised methods [28], in v-bottom plates (Greiner, Dungannon, UK) using a 0.75% *v*/*v* suspension of turkey red blood cells in PBS and 4 haemagglutination units of virus. RDE-treated human serum samples were used directly in the assay, with a starting dilution of 1:10. Titres were tabulated as the reciprocal of the end-point titre.

### 2.4. Microneutralisation (MN) Assay

Microneutralisation assays were carried out using the method described by Lin et al. [29] using MDCK-SIAT1 cells with a starting serum dilution of 1:10. Results were analysed using a custom Labview software, and the end-point titre was determined as the titre of sera at which a 50% reduction in the infection of cells was observed. Titres were tabulated as the reciprocal of the end-point titre.

### 2.5. Statistical Analysis

Raw data for HI and MN assays were tabulated in Microsoft Excel (Office 2019). Non-normal distribution of data was established by plotting distribution curves in GraphPad Prism (version 10). To account for non-normal distribution, the Wilcoxon matched-pairs signed-rank test was used to measure the significance of the difference between the means of paired pre- and post-vaccination serum panels in GraphPad Prism (version 10). Geometric Mean Titres (GMT), 95% confidence intervals of the GMT, percentage of titres ≥ 40, mean fold rise and percentage of volunteers with a rise in antibody titre post-vaccination were calculated for each year using custom software and GraphPad Prism (version 10). Titres below the detection threshold of 10 were converted to a nominal titre of 5 for log transformation. To investigate the necessity of administering booster doses of vaccine to the population in the event of a swine influenza outbreak in humans, the waning of antibody titres between post-vaccination one year and pre-vaccination the following year was investigated using microneutralisation data. Collected data was refined to select for volunteers who returned for at least three years in a row. Data was used for the years between 2021 and 2024, when volunteers returned most consistently. GMTs and 95% confidence intervals of the GMT were calculated using GraphPad Prism (version 10). All analyses and graphs were built in GraphPad Prism (version 10).

## 3. Results

To understand the ability of the seasonal influenza vaccine to induce cross-reactive antibodies against currently circulating swine influenza A viruses, we tested representative viruses from five different clades in HI and MN assays using pre- and post-vaccination human sera panels. Table 2 summarises these results. High post-vaccination GMTs were observed for the vaccine strain virus in all years by both HI and MN. Though pre-vaccination GMTs were also often above protective levels, a significant difference between pre- and post-vaccination titres was observed each year by both HI and MN against the vaccine strain viruses. An alignment of HA protein sequences for the five SwIAV viruses can be found in Supplementary Information (Supplementary Figure S1).

**Figure 1:**
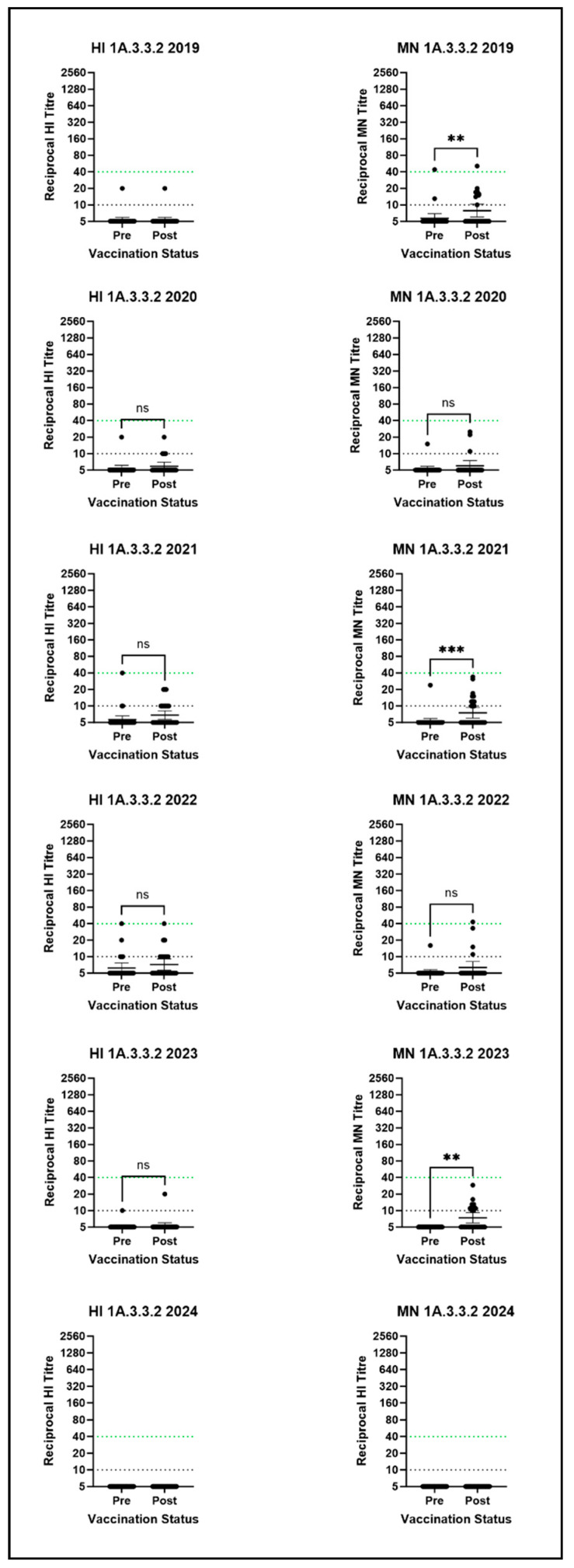
HI and MN assay results for the 1A.3.3.2 clade virus against all six human serum panels. Stars denote significance between pre- and post-vaccination titres as calculated by the Wilcoxon test: ** = *p* ≤ 0.01; *** = *p* ≤ 0.001; ns = *p* > 0.05. Points represent individual titres and horizontal lines denote the GMT and 95% CI. The dotted green line represents the CoP, and the dotted black line is the detection threshold. N.B there is no established correlate of protection for the MN assay.

We observed heterogeneity in cross-reactive responses for most clades of SwIAV tested, by assay and by vaccine year. Interestingly, although the 1A.3.3.2 lineage in pigs arose from the A/H1N1 pdm09 viruses in humans by reverse zoonosis, no vaccine-induced cross-reactive antibodies were observed against the 1A.3.3.2 virus by either HI or MN (Figure 1), suggesting very low levels of pre-existing cross-reactive antibodies that were not boosted by seasonal vaccination. Pre- and post-vaccination 1A.3.3.2 virus GMTs were below the detection threshold in all years, and no significant differences between pre- and post-vaccination titres were observed in any serum panel by HI. Minimal to no post-vaccination fold-rises in antibody titre were seen for this clade of virus by HI (Supplementary Figure S2; although by MN, an antibody titre rise was observed in a maximum of 42% of participants (in 2023) and a minimum of 0% of participants (in 2024), as seen in Supplementary Figure S3. This lack of recognition for the current 1A.3.3.2 viruses in pigs is likely a result of differential antigenic drift in both humans and pigs, where over time the potential for 1A.3.3.2 viruses to be recognised by human immunity to current A/H1N1/pdm09 viruses is lost.

**Figure 2:**
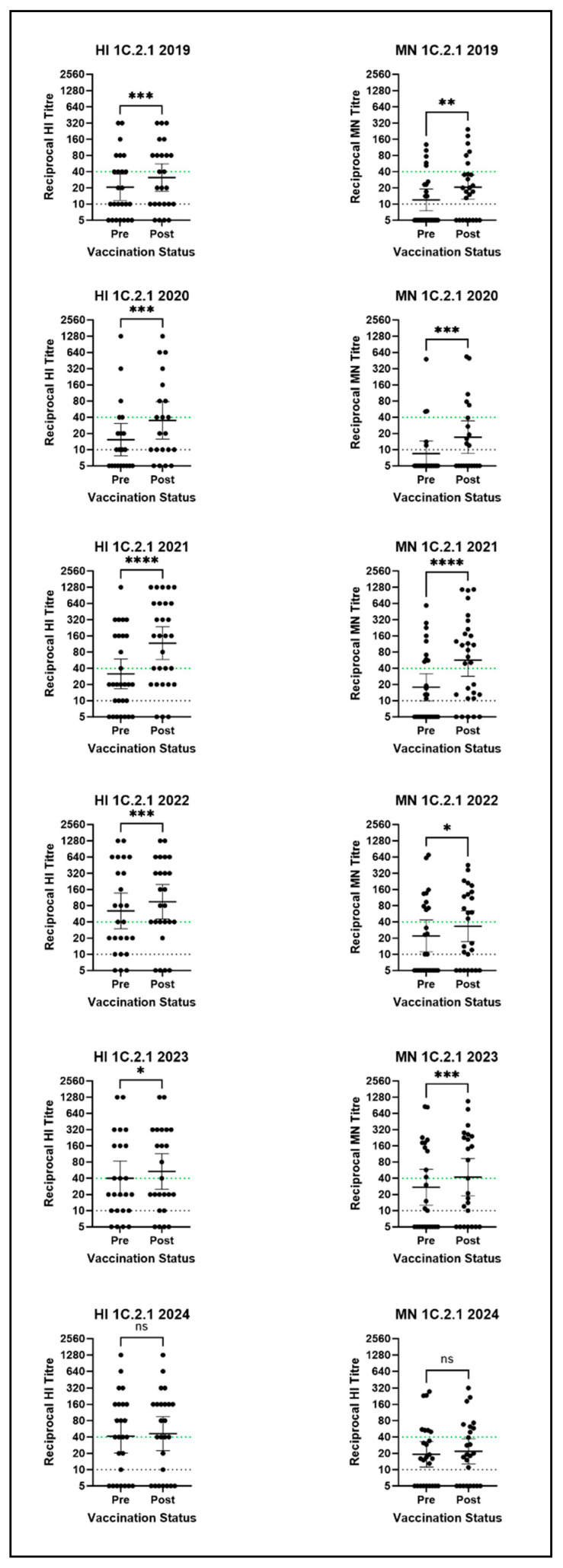
HI and MN assay results for the 1C.2.1 clade virus against all six human serum panels. Stars denote significance between pre- and post-vaccination titres as calculated by the Wilcoxon test: * = *p* ≤ 0.05; ** = *p* ≤ 0.01; *** = *p* ≤ 0.001; **** = *p* ≤ 0.0001; ns = *p* > 0.05. Points represent individual titres and horizontal lines denote the GMT and 95% CI. The dotted green line represents the CoP, and the dotted black line is the detection threshold. N.B there is no established correlate of protection for the MN assay.

**Figure 3:**
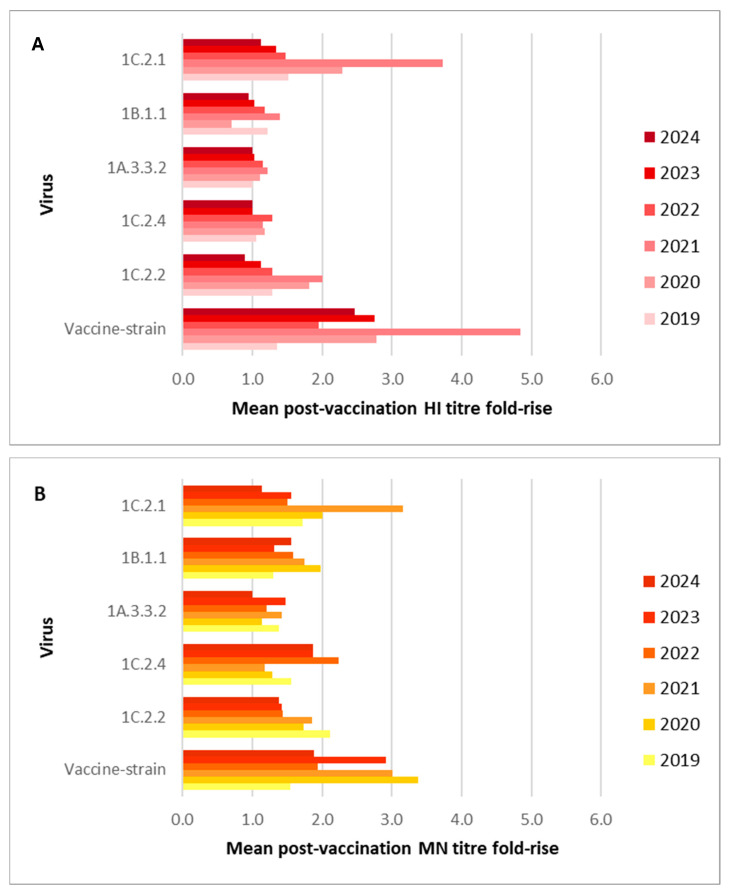
The mean fold-rise in HI (**A**) and MN (**B**) titres against five different clades of swine virus and the respective vaccine strain, following vaccination with the seasonal influenza vaccine across six years.

Seasonal influenza vaccine-induced antibodies were, however, observed against representative viruses from other SwIAV clades, particularly for the representative 1C.2.1 virus. As seen in Figure 2, post-seasonal vaccination GMTs against the 1C.2.1 virus were ≥40 in four out of six years by HI and in two out of six years by MN. This suggests the seasonal vaccine induced cross-reactive antibodies against this clade of SwIAV, which met the correlate of protection in four out of six years. A mean 4-fold rise in post-vaccination titres was not seen in any years for any SwIAV; however, the 1C.2.1 virus had similar fold-rises as the vaccine-strain virus in many years by both HI and MN, as seen in Figure 3. Between 44% and 79% of volunteers had a rise in cross-reactive antibody titre against the 1C.2.1 virus measured by MN and between 16% and 76% by HI following vaccination with the seasonal influenza vaccine. Further, increases in post-vaccination GMTs compared to pre-vaccination GMTs were observed each year by both methods. A high percentage of volunteers with post-vaccination HI and MN titres ≥ 40 was observed each year: between 48% and 80% by HI and 24% and 59% by MN. Statistically significant increases in titres were observed each year following seasonal vaccination, except in 2024, by both HI (2019: *p* = 0.0010, 2020: *p* = 0.0002, 2021: *p* < 0.0001, 2022: *p* = 0.0005, 2023: *p* = 0.0156) and MN (2019: *p* = 0.0016, 2020: *p* = 0.001, 2021: *p* < 0.0001, 2022: *p* = 0.026 and 2023: *p* = 0.0008). Collectively, these results suggest the seasonal vaccine induces cross-reactive antibodies against the currently circulating 1C.2.1 clade of swine influenza A virus.

**Figure 4:**
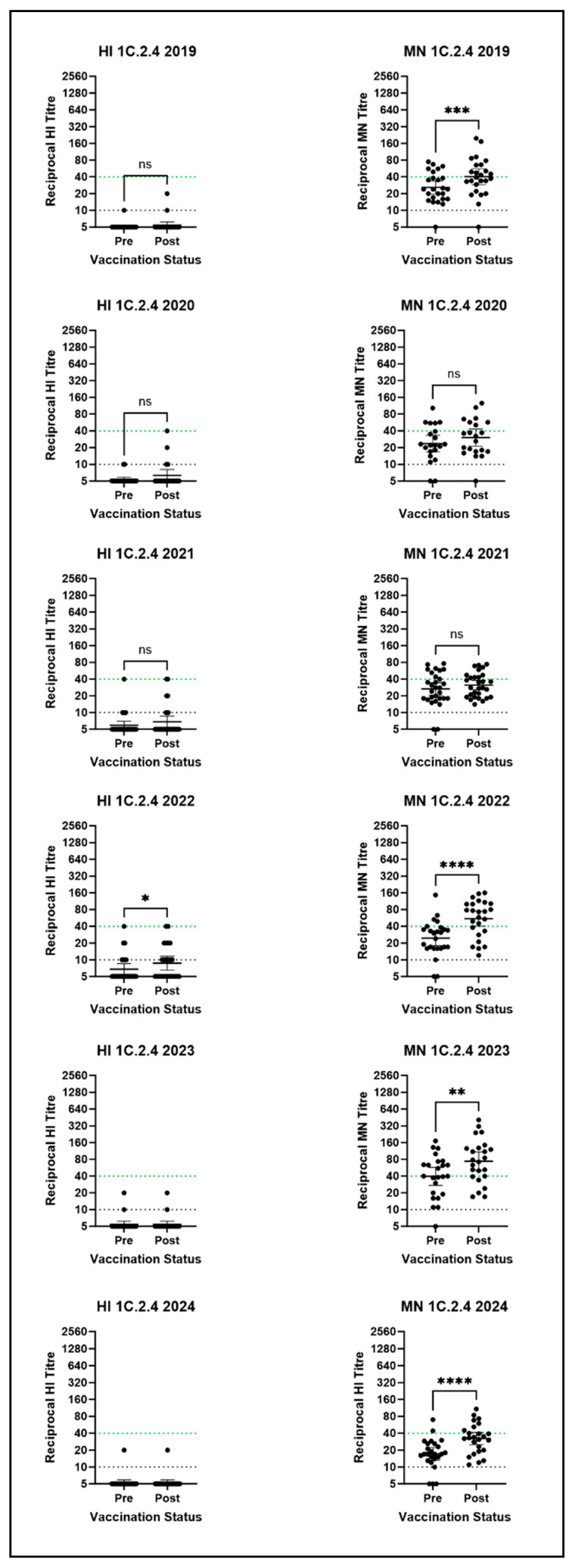
HI and MN assay results for the 1C.2.4 clade virus against all six human serum panels. Stars denote significance between pre- and post-vaccination titres as calculated by the Wilcoxon test: * = *p* ≤ 0.05; ** = *p* ≤ 0.01; *** = *p* ≤ 0.001; **** = *p* ≤ 0.0001; ns = *p* > 0.05. Points represent individual titres and horizontal lines denote the GMT and 95% CI. The dotted green line represents the CoP, and the dotted black line is the detection threshold. N.B there is no established correlate of protection for the MN assay.

A discrepancy was observed between the antibody titres measured by HI and MN against the 1C.2.4 virus, with MN data suggesting a far greater response to seasonal influenza vaccination than HI data (Figure 4). Increases in the percentage of post-vaccination MN titres ≥ 40 were observed against the representative 1C.2.4 virus in all years, with a significant difference between pre- and post-vaccination titres in four out of six serum panels (2019: *p =* 0.0002, 2022: *p* < 0.0001, 2023: *p =* 0.0052, and 2024: *p* < 0.0001). Post-seasonal influenza vaccination MN titres ≥ 40 peaked at 75% in 2023, rising from 54% pre-vaccination, depicted in Supplementary Figure S4. A notable rise in MN titres ≥ 40 was observed in 2022: 68% post-vaccination compared to 20% pre-vaccination. GMTs ≥ 40 were observed in three out of six years for the 1C.2.4 virus by MN. These results suggest the seasonal vaccine is able to induce cross-reactive antibodies against the representative 1C.2.4 virus and may be of utility in the event of an outbreak of this clade of virus in humans. However, HI results did not reflect MN results and do not suggest that the seasonal vaccine induces cross-reactive antibodies against this clade. This may be due to differences in the type of antibody response detected by the two assays; HI assays detect antibodies that bind to the globular head of the HA, preventing binding of the HA to the red blood cells used in the assay, whereas the MN detects all neutralising antibodies, which include those binding to the globular head of the HA, but also potentially other regions of the HA, such as the stem region, as well as any neutralising antibody response to the neuraminidase (NA). Post-vaccination GMTs below the HI assay detection threshold were observed each year with minimal fold-rises seen in Figure 3A. No or very low numbers of post-vaccination titres ≥ 40 were observed each year by HI (Supplementary Figure S4).

**Figure 5:**
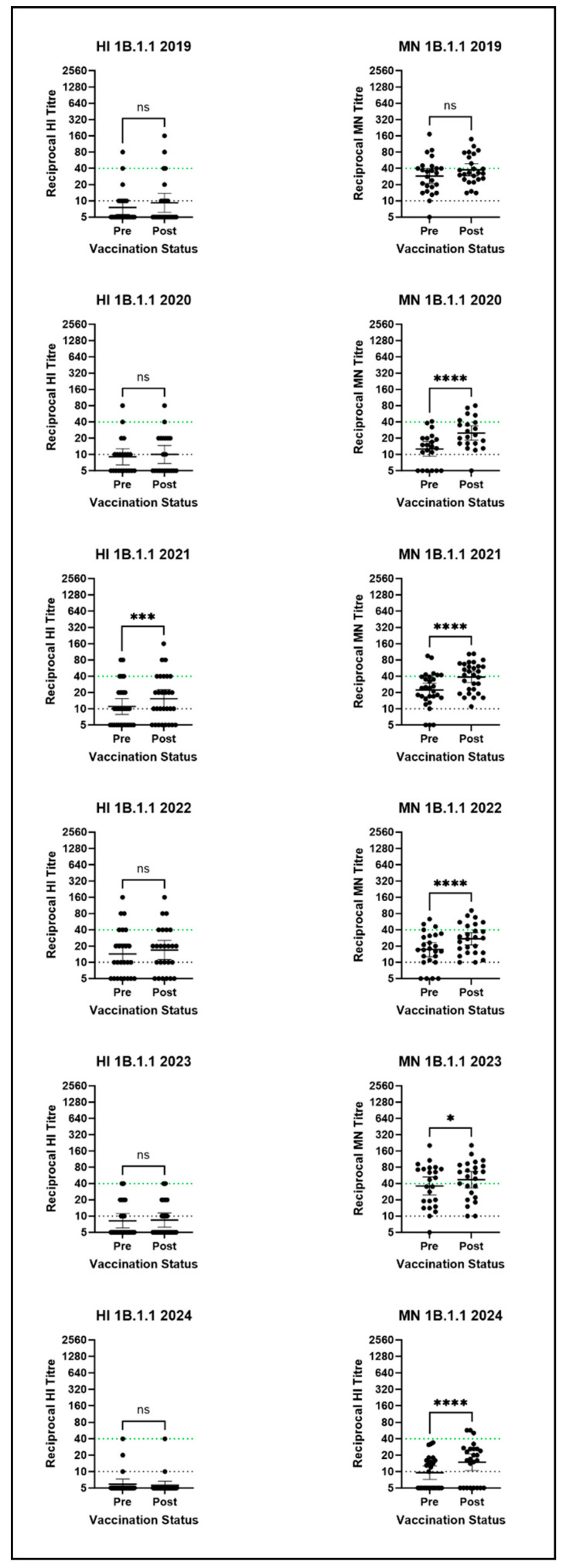
HI and MN assay results for the 1B.1.1 clade virus against all six human serum panels. Stars denote significance between pre- and post-vaccination titres as calculated by the Wilcoxon test: * = *p* ≤ 0.05; *** = *p* ≤ 0.001; **** = *p* ≤ 0.0001; ns = *p* > 0.05. Points represent individual titres and horizontal lines denote the GMT and 95% CI. The dotted green line represents the CoP, and the dotted black line is the detection threshold. N.B there is no established correlate of protection for the MN assay.

Similarly to the 1C.2.4 clade virus, much lower post-seasonal vaccination antibody titres were observed by HI against the representative 1B.1.1 clade SwIAV than by MN (Figure 5). The post-vaccination MN GMT of 47 in 2023 was the only GMT ≥ 40 for the 1B.1.1 virus, and a correlate of protection has not been established for MN titres. However, an increase in the percentage of titres ≥ 40 post-vaccination was observed in five out of six years by MN, reaching 63% of post-vaccination titres in 2023. Additionally, a rise in antibody titre against the 1B.1.1 virus between pre- and post-vaccination was observed in 64% to 86% of volunteers by MN, suggesting the seasonal vaccine induces cross-reactive antibodies against this virus, which fail to reach the higher titres observed for the 1C.2.1 virus. Further, a significant difference between pre- and post-vaccination MN titres was observed in five out of six serum panels (2020: *p* < 0.0001; 2021: *p* < 0.0001; 2022: *p* = 0.0001; 2023: *p* = 0.0191; and 2024: *p* = 0.0001), as seen in Figure 5. However, by HI, minimal or no rises in post-vaccination titres ≥ 40 were observed, and post-vaccination GMTs were low or below detection thresholds. Therefore, HI assay results do not support the results observed in the MN assay and do not indicate that the seasonal influenza vaccine is able to induce any cross-reactive antibodies against the 1B.1.1 clade SwIAV. This difference is likely due to the different antibody responses measured by the two assays [29].

**Figure 6:**
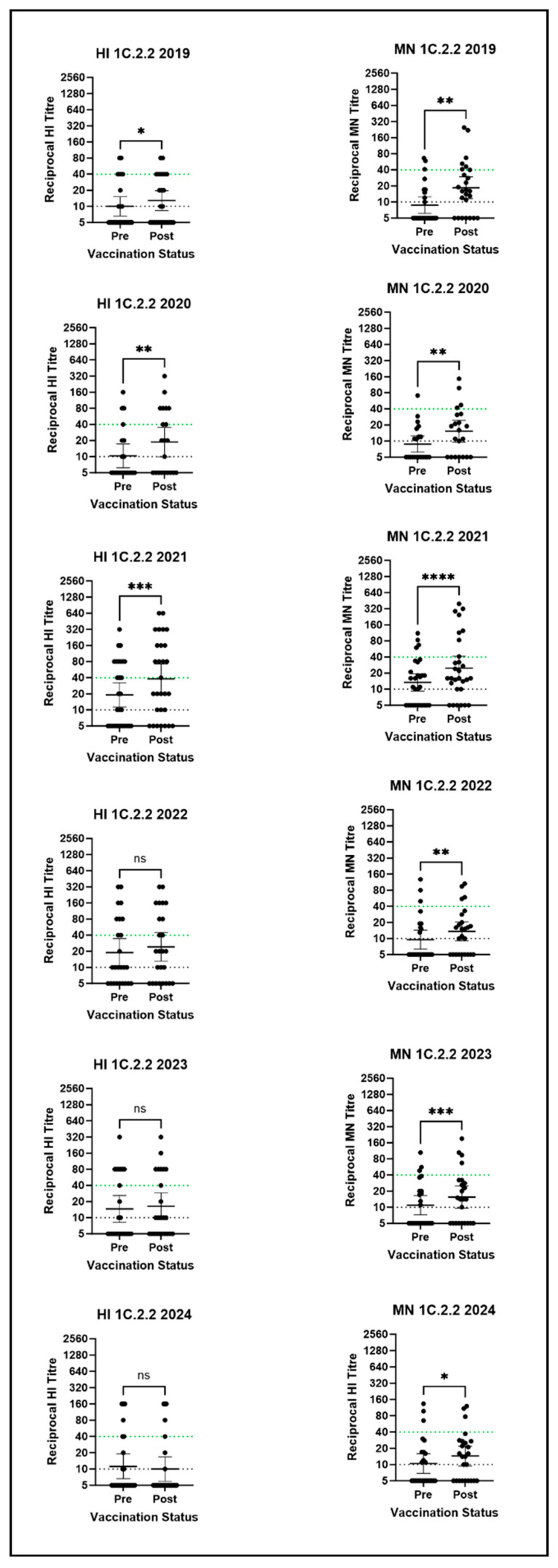
HI and MN assay results for the 1C.2.2 clade virus against all six human serum panels. Stars denote significance between pre- and post-vaccination titres as calculated by the Wilcoxon test: * = *p* ≤ 0.05; ** = *p* ≤ 0.01; *** = *p* ≤ 0.001; **** = *p* ≤ 0.0001; ns = *p* > 0.05. Points represent individual titres and horizontal lines denote the GMT and 95% CI. The dotted green line represents the Correlate of Protection (CoP), and the dotted black line is the detection threshold. N.B there is no established correlate of protection for the MN assay.

Post-seasonal vaccination GMTs for the 1C.2.2 representative virus did not meet the correlate of protection in any year by HI and were low by MN in all years (Figure 6). However, though the percentage of volunteers with a rise in post-seasonal vaccination HI titre varied greatly by year (ranging from 4 to 48%), a rise in post-vaccination MN titre was observed in between 52% and 66% of volunteers. Further, a significant difference between pre- and post-seasonal vaccination MN titres was observed each year (2019: *p* = 0.0038, 2020: *p* = 0.0016, 2021: *p* < 0.0001, 2022: *p* = 0.0096, 2023: *p* = 0.0004 and 2024: *p* = 0.0125) and in three out of six years by HI (2019: *p* = 0.0312, 2020: *p* = 0.0078 and 2024: *p* = 0.0001). This suggests that the seasonal vaccine was able to induce cross-reactive antibodies, which failed to reach protective levels. This is reflected in the low percentage of volunteers with post-seasonal vaccination HI and MN titres ≥ 40, with little difference when compared to pre-vaccination HI and MN titres ≥ 40.

### 3.1. Low Pre-Vaccination Titres

**Figure 7:**
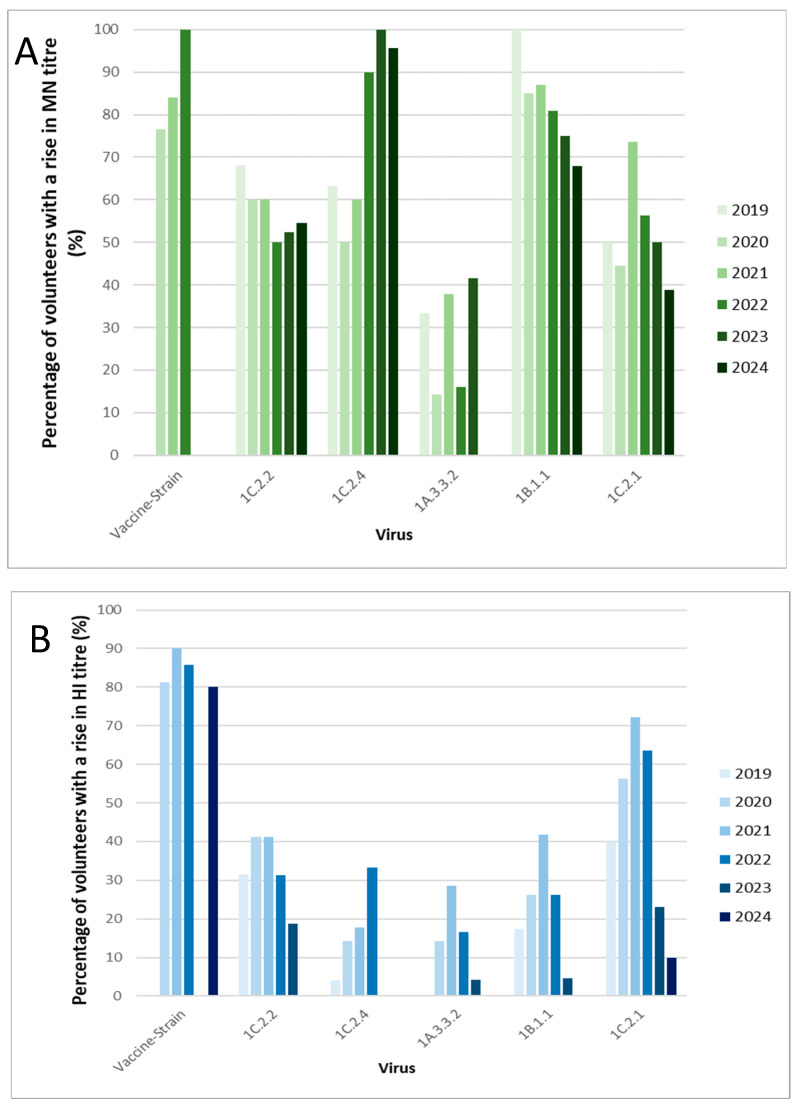
The percentage of donors, with pre-vaccination titres less than 40, who had a rise in HI (**A**) or MN (**B**) titre against the respective vaccine strain and five different clades of swine influenza A virus following vaccination with the seasonal influenza vaccine across six years. Please note that in 2019 and 2023, there were no donors with pre-vaccination HI titres less than 40 against the respective vaccine strains. The absence of a bar in all other instances is reflective of zero percent of the donors with a rise in HI titre.

When adjusting analyses to incorporate only volunteers with pre-vaccination titres below 40, a higher percentage of volunteers with an rise in post-vaccination titre was observed in five out of six years for the 1B.1.1 virus by MN. The percentage of volunteers with low pre-vaccination titres who had a rise in MN titres against this clade ranged between 75% and 100%, as can be seen in Figure 7A. Similarly, a higher percentage of volunteers with a rise in antibody titre by MN was observed for the 1C.2.4 virus, with a range of 50% to 100%. By HI, however (Figure 7B), the percentage of volunteers with a rise in antibody was similar compared to all volunteers for most clades of virus. No notable increases in fold-changes were observed by HI or MN when comparing volunteers with low pre-vaccination titres to the whole cohort (Supplementary Figures S5 and S6). Overall, post vaccination GMTs (Supplementary Figures S7–S11) and the percentage of post-vaccination titres ≥ 40 (Supplementary Figures S12 and S13) were lower in volunteers with pre-vaccination titres < 40 compared to all volunteers. Serum panel sizes adjusted to include only volunteers with pre-vaccination titres below 40 can be seen in Supplemental Tables S2 and S3 for the HI assay and MN assay respectively.

### 3.2. Waning Immunity

**Figure 8:**
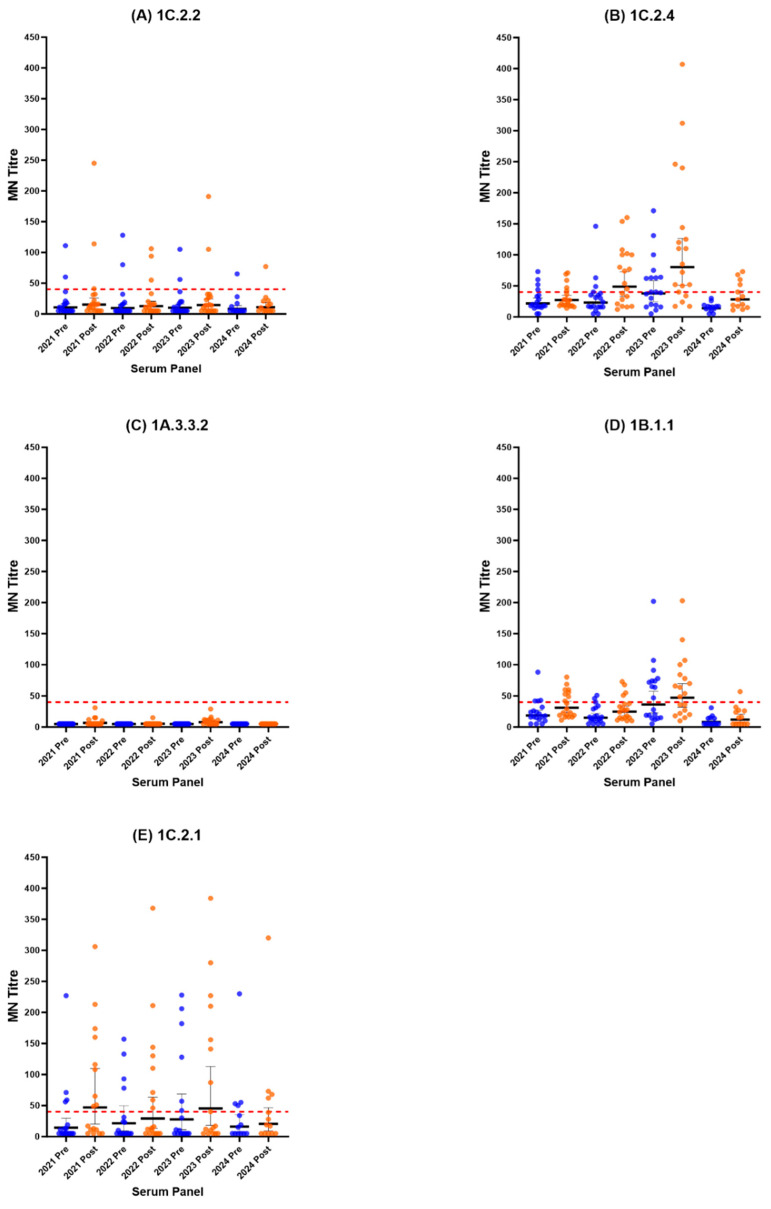
Post-vaccination (in orange) and pre-vaccination (in blue) microneutralisation assay titres and GMTs (black bold lines) calculated for data obtained from participants who returned for three consecutive years, 2021-2023 (*n* = 19). (**A**) Consecutive GMTs for serum samples tested against the 1C.2.2 virus. (**B**) Consecutive GMTs for serum samples tested against the 1C.2.4 virus. (**C**) Consecutive GMTs for serum samples tested against the 1A.3.3.2 virus. (**D**) Consecutive GMTs for serum samples tested against the 1B.1.1 virus. (**E**) Consecutive GMTs for serum samples tested against the 1C.2.1 virus.

Microneutralisation data for volunteers who returned each year between 2021 and 2024 (*n* = 19) were used to understand the waning of antibody titres between post-vaccination and pre-vaccination, and are represented in Figure 8. This is a small subset, and as such, the statistical power of this analysis is limited; therefore, conclusions must be viewed with this in mind. A rise in antibody titres post-vaccination was observed against most SwIAV clades, except 1A.3.3.2 and very minimally against the 1C.2.2 virus. This rise in titres was followed by a fall in antibody titres by the following year, showing the waning of vaccine-induced antibodies. This suggests that, in the event of a swine influenza outbreak in humans, a booster dose of vaccine for those who have already received the vaccine may be necessary to boost antibody titres that may have waned. However, in the event of a swine influenza outbreak caused by a 1A.3.3.2, a booster dose of vaccine is unlikely to be of utility due to very low post-vaccination antibody titres.

## 4. Discussion

SwIAV can be transmitted between pigs and humans and has the potential to cause influenza outbreaks or pandemics in the human population. Influenza vaccination is the most effective method for preventing influenza infections, and seasonal vaccines are generally readily available. Therefore, the ability of the seasonal influenza vaccine to generate cross-reactive antibodies against SwIAV was investigated by testing pre- and post-vaccination human sera panels against representative viruses for different SwIAV clades by haemagglutination inhibition assay and microneutralisation assay.

A clade-dependent boosting of antibody titres, which often failed to reach the established correlate of protection, was observed. As expected, most people did not have pre-vaccination titres ≥ 40 against any of the swine influenza A viruses, indicating poor baseline population immunity. Vaccine administration resulted in a titre-boost for most clades of virus, more significantly for some than others. For instance, significant differences between pre- and post-vaccination titres were observed each year by MN for the 1C.2.2 virus, and by both assays for the 1C.2.1 virus (except in 2024). Additionally, high post-vaccination GMTs and percentages of participants with an increase in antibody titre were observed for the 1C.2.1 virus. Therefore, both HI and MN results suggest that the seasonal influenza vaccine is likely to be beneficial in the event of a SwIAV 1C.2.1 clade outbreak. Further, the boosting and waning pattern seen for the 1C.2.1 virus suggests that a booster dose of vaccine for the vaccinated is likely to boost antibody titres that may have waned. However, overall results for the 1C.2.2 virus were less encouraging, with low GMTs and proportions of post-vaccination titres ≥ 40. Therefore, it is unlikely that the seasonal vaccine will offer protection against 1C.2.2 viruses.

A significant boosting of titres was not observed for the 1A.3.3.2 virus by HI assay and only in three years by MN assay, which, coupled with very low GMTs, suggests that the vaccine does not induce cross-reactive antibodies against this clade of virus. This, in conjunction with very low pre-vaccination GMTs, highlights that viruses belonging to this clade may be a particular public health threat, and readiness to produce specific vaccines against these viruses should be reviewed. These results are at odds with those previously published using both ferret models and human serology, which showed that the vaccine protected ferrets against challenge with 1A lineage SwIAV and produced high antibody titres against 1A lineage viruses in humans [21,23]. The differences observed may be due to antigenic differences between the viruses used and differences in vaccine strains. For example, in the paper by van Diemen et al., the authors used a vaccine containing the A/California/7/2009 strain of H1N1 from the 1A.3.3.2 pandemic lineage. It is reasonable to assume that this will be more antigenically similar to swine viruses than the more recent vaccine strains (from 2019 to 2022) used in the vaccines included in this study. Additionally, in vivo challenge studies in ferrets may be influenced by responses not detected by HI or MN, such as non-neutralising antibodies or cell-mediated immunity.

Overall, results from the MN assay support a better vaccine-induced cross-reactive antibody response. In particular, MN data suggest that the vaccine induces some degree of cross-reactive antibodies against the representative 1B.1.1 and 1C.2.4 viruses, whereas HI data do not support this. A correlate of protection has not been established for MN assay antibody titres, and methods used vary greatly. However, the MN assay may be able to measure the presence of functional neutralising antibodies that not only bind to the globular head of the HA, but also antibodies binding the stem region of the influenza haemagglutinin glycoprotein and the neuraminidase glycoprotein (as opposed to antibodies which prevent the binding of influenza virus to red blood cells) and may therefore be a better reflection of protective antibodies [29]. Where results show a low HI titre but high MN titre, we would suggest that this is indicative of a biologically relevant response that is via functionally neutralising antibodies that are not detected in the HI assay.

Variation in antibody titres was also seen between years; for instance, the microneutralisation GMT for the 1C.2.1 virus ranged from 17 in 2020 to 56 in 2021. Volunteer panels were not identical each year and were small in size, which may have resulted in the observed inter-season variation. Furthermore, seasonal influenza vaccine antigens are reviewed and regularly updated due to antigenic drift. While collecting the serum panels used here, four different H1N1pdm09 antigens were used in influenza vaccinations. Variation in cross-reactive responses to SwIAV may be due to the differing ability of vaccine antigens to induce cross-protective antibodies. Sequential annual vaccination with different influenza antigens has been shown to induce higher levels of cross-reactive antibodies to heterologous seasonal H1N1, H3N2 and influenza B strains when compared to sequential annual vaccination with the same influenza antigen [30]. Due to the changing cohort used in this study, no conclusions about the effect of changing vaccine formulations can be made.

When adjusting analyses to include only those volunteers with pre-vaccination HI and MN titres < 40, minimal differences were seen for the most part. However, the improved antibody response against the 1B.1.1 virus suggests that vaccination of those likely to have low pre-vaccination titres, such as the unvaccinated, may be beneficial in response to an outbreak. Again, differences between the entire cohort and those with low pre-vaccination titres were only observed by MN and may be related to the type of antibody response stimulated [29]. Overall, post-vaccination GMTs were lower in people with pre-vaccination titres < 40.

A systematic review found that post-influenza vaccination antibody levels waned within six months but remained higher than pre-vaccination titres [31]. The administration of a booster dose of vaccine in the event of a swine influenza outbreak in humans is likely to help boost antibody titres in those who have previously been vaccinated, except for the 1A.3.3.2 virus. However, only two time points (almost one year apart) are represented here, and therefore, conclusions about antibody titres at other time points cannot be made. Additionally, volunteers did not report influenza infections occurring during this time period, which could reduce the appearance of waning immunity in the cohort.

These results suggest that the seasonal vaccine does not induce cross-reactive antibodies against all lineages, or even all clades within a lineage, of swine H1 influenza A viruses. The value of the seasonal vaccine in the event of a swine influenza outbreak will be dependent on the strain of virus, as will the benefit of administering a booster dose to those who have already been vaccinated. However, due to the availability of the seasonal vaccine, its use should be considered in response to a swine influenza outbreak while a strain-specific vaccine is produced. Continued surveillance of SwIAV and development of candidate vaccine viruses is vital due to low population immunity in humans. Moreover, these results highlight the need for the continued promotion of influenza vaccination due to its ability to induce cross-reactive antibodies against some H1 swine influenza viruses.

## Figures and Tables

**Table 1:** H1N1pdm09 vaccine strain viruses and swine influenza A viruses used. The year in which the vaccine strain was included in the northern hemisphere vaccine is provided, as well as lineage and clade information for swine viruses.

Vaccine Strain	Influenza Season	Accession Number
IVR-190 (A/Brisbane/02/2018) (Egg-grown)	2019–20	EPI_ISL_20212507
A/Guangdong-Maonan/SWL1536/2019 (Egg-grown)	2020–21	EPI_ISL_18991164
A/Wisconsin/588/2019 (Cell-grown)	2021–22, 2022–23	EPI_ISL_19085699
IVR-238 (A/Victoria/4897/2022) (Egg-grown)	2023–24, 2024–25	EPI_ISL_19828785
**Swine Viruses**	**Clade**	
A/swine/Italy/25675-1/2022	1C.2.2	EPI_ISL_19644598
A/swine/Italy/49701-6/2022	1C.2.4	EPI_ISL_14937237
A/swine/Italy/262599-4/2022	1A.3.3.2	EPI_ISL_19644608
A/swine/England/045393/2022	1B.1.1	EPI_ISL_19880217
A/swine/Italy/133898-3/2022	1C.2.1	EPI_ISL_19644565

**Table 2:** Summary of HI and MN results for each SwIAV clade by year, showing the percentage of volunteers with post-vaccination titres ≥ 40, percentage of volunteers with a rise in titre post-vaccination, post-vaccination GMT and a comment about whether this indicates a seasonal vaccine boost would be useful. ND is used in place of a *p*-value where no difference was observed between pre- and post-vaccination titres.

Year	HI				MN				Seasonal Vaccine of Use?
	Post-Vacc % HI Titres ≥ 40	Percentage of Volunteers with Rise in Titre (%)	Post-Vacc GMT	Pre vs. Post *p* Value	Post-Vacc % MN Titres ≥ 40	Percentage of Volunteers with Rise in Titre (%)	Post-Vacc GMT	Pre vs. Post *p* Value	
**Vaccine-strain**									
2019	92	32	139	0.0195	88	76	309	0.0002	Yes
2020	57	67	41	0.0001	52	76	58	<0.0001	Yes
2021	90	86	213	<0.0001	69	86	65	<0.0001	Yes
2022	92	76	106	0.0023	52	92	48	<0.0001	Yes
2023	96	75	196	0.0004	100	100	428	<0.0001	Yes
2024	88	48	196	0.0182	100	84	479	<0.0001	Yes
**1C.2.2**									
2019	32	24	13	0.0312	21	64	18	0.0038	No
2020	38	38	19	0.0078	19	62	15	0.0016	No
2021	48	48	38	0.0001	28	66	25	<0.0001	Maybe
2022	40	36	24	0.0527	16	52	14	0.0096	No
2023	33	17	16	0.1250	17	58	15	0.0004	No
2024	20	4	10	0.7500	12	56	14	0.0125	No
**1C.2.4**									
2019	0	4	5	>0.9999	48	68	40	0.0002	Maybe
2020	5	14	6	0.2500	33	48	30	0.4610	No
2021	7	17	7	0.0625	34	48	31	0.3829	No
2022	8	32	9	0.0234	68	84	55	<0.0001	Maybe
2023	0	0	5	ND	75	75	74	0.0052	Maybe
2024	0	0	5	ND	32	96	32	<0.0001	Maybe
**1A.3.3.2**									
2019	0	0	5	ND	4	36	8	0.0039	No
2020	0	14	6	0.2500	0	14	6	0.2500	No
2021	0	28	7	0.0977	0	38	8	0.0010	No
2022	4	16	7	0.1250	4	16	6	0.1250	No
2023	0	4	5	>0.9999	0	42	7	0.0020	No
2024	0	0	5	ND	0	0	5	ND	No
**1B.1.1**									
2019	16	16	9	0.1250	32	64	37	0.3026	No
2020	10	24	6	0.1406	24	86	25	<0.0001	Maybe
2021	28	41	15	0.0005	55	83	39	<0.0001	Maybe
2022	28	20	17	0.0625	28	80	27	<0.0001	Maybe
2023	8	4	8	>0.9999	63	71	47	0.0191	Maybe
2024	4	0	6	>0.9999	12	68	15	<0.0001	No
**1C.2.1**									
2019	48	44	31	0.0010	24	56	21	0.0016	Maybe
2020	48	62	35	0.0002	24	52	17	0.0010	Maybe
2021	72	76	117	<0.0001	59	79	56	<0.0001	Yes
2022	80	48	94	0.0005	52	64	33	0.0260	Yes
2023	50	29	53	0.0156	50	67	42	0.0008	Yes
2024	64	16	46	0.1250	32	44	22	0.1262	Maybe

## Data Availability

The raw data supporting the conclusions of this article will be made available by the authors on request.

## Supplementary Materials

The following supporting information can be downloaded at: https://www.mdpi.com/article/10.3390/vaccines14030211/s1, Figure S1: Alignment of HA protein sequences for the panel of SwIAV used in this study compared to A/Wisconsin/588/2019. Accession numbers for each virus can be found in Table 1; Figure S2: The percentage of donors with a rise in HI titre against the respective vaccine strain and five different clades of swine influenza A virus, following vaccination with the seasonal influenza vaccine across six years; Figure S3: The percentage of donors with a rise in MN titre against the respective vaccine strain and five different clades of swine influenza A virus, following vaccination with the seasonal influenza vaccine across six years; Figure S4: The percentage of HI (panel A) and MN (panel B) assay titres greater than 40 by serum panel. All five clades of swine virus and the relevant vaccine strain are included. Note, an MN titre of 40 is used as a guide and is not an established correlate of protection; Figure S5: The mean fold-rise in HI titres for donors with pre-vaccination titres less than 40 against five different clades of swine virus and the respective vaccine strain, following vaccination with the seasonal influenza vaccine across six years. The vaccine strain is excluded where no donors had pre-vaccination MN titres less than 40; Figure S6: The mean fold-rise in MN titres for donors with pre-vaccination titres less than 40 against five different clades of swine virus and the respective vaccine strain, following vaccination with the seasonal influenza vaccine across six years. The vaccine strain is excluded where no donors had pre-vaccination MN titres less than 40; Figure S7: HI and MN assay results, using only donors with pre-vaccination titres less than 40, against the 1C.2.2 clade virus against all six human serum panels. Stars denote significance between pre- and post-vaccination titres as calculated by the Wilcoxon test: * = *p* ≤ 0.05; ** = *p* ≤ 0.01; *** = *p* ≤ 0.001; ns = *p* > 0.05. Points represent individual titres and horizontal lines denote the GMT and 95% CI. The dotted green line represents the CoP and the dotted black line is the detection threshold; Figure S8: HI and MN assay results, using only donors with pre-vaccination titres less than 40, against the 1C.2.4 clade virus against all six human serum panels. Stars denote significance between pre- and post-vaccination titres as calculated by the Wilcoxon test: * = *p* ≤ 0.05; *** = *p* ≤ 0.001; **** = *p* ≤ 0.0001; ns = *p* > 0.05. Points represent individual titres and horizontal lines denote the GMT and 95% CI. The dotted green line represents the CoP and the dotted black line is the detection threshold; Figure S9: HI and MN assay results, using only donors with pre-vaccination titres less than 40, against the 1A.3.3.2 clade virus against all six human serum panels. Stars denote significance between pre- and post-vaccination titres as calculated by the Wilcoxon test: ** = *p* ≤ 0.01; *** = *p* ≤ 0.001; ns = *p* > 0.05. Points represent individual titres and horizontal lines denote the GMT and 95% CI. The dotted green line represents the CoP and the dotted black line is the detection threshold; Figure S10: HI and MN assay results, using only donors with pre-vaccination titres less than 40, against the 1B.1.1 clade virus against all six human serum panels. Stars denote significance between pre- and post-vaccination titres as calculated by the Wilcoxon test: ** = *p* ≤ 0.01; *** = *p* ≤ 0.001; **** = *p* ≤ 0.0001; ns = *p* > 0.05. Points represent individual titres and horizontal lines denote the GMT and 95% CI. The dotted green line represents the CoP and the dotted black line is the detection threshold; Figure S11: HI and MN assay results, using only donors with pre-vaccination titres less than 40, against the 1C.2.1 clade virus against all six human serum panels. Stars denote significance between pre- and post-vaccination titres as calculated by the Wilcoxon test: * = *p* ≤ 0.05; ** = *p* ≤ 0.01; *** = *p* ≤ 0.001; ns = *p* > 0.05. Points represent individual titres and horizontal lines denote the GMT and 95% CI. The dotted green line represents the CoP and the dotted black line is the detection threshold; Figure S12: The percentage of post-vaccination HI assay titres greater than or equal to 40, the correlate of protection, by serum panel, adjusted to include only donors with pre-vaccination titres less than 40. All five clades of swine virus and the relevant vaccine strain are included. The vaccine strain is excluded where no donors had pre-vaccination HI titres less than 40; Figure S13: The percentage of post-vaccination MN assay titres greater than or equal to 40 by serum panel, adjusted to include only donors with pre-vaccination titres less than 40. All five clades of swine virus and the relevant vaccine strain are included. The vaccine strain is excluded where no donors had pre-vaccination MN titres less than 40; Table S1: Serum panel sizes; Table S2: Serum panel sizes tested by HI when adjusted to include only donors with pre-vaccination titres less than 40 against the respective viruses; Table S3: Serum panel sizes tested by MN when adjusted to include only donors with pre-vaccination titres less than 40 against the respective viruses.

## Abbreviations

The following abbreviations are used in this manuscript:

IAV	Influenza A Viruses
SwIAV	Swine influenza A virus
DMEM + PS	DMEM containing 5% penicillin and streptomycin
HI	Haemagglutination Inhibition
MN	Microneutralisation
GMT	Geometric Mean Titre
CoP	Correlate of Protection
