# Supplementary material for: Investigation of Potential Cross-Protection Conferred by the Seasonal Influenza Vaccine Against Swine Influenza A Viruses of Pandemic Potential
Source: Vaccines (Basel). 2026 Feb 26;14(3):211. doi: 10.3390/vaccines14030211 (PMC13030337; doi:10.3390/vaccines14030211)
Supplement: Supplementary file 1 [file vaccines-14-00211-s001.zip › vaccines-4078394-supplementary.pdf]

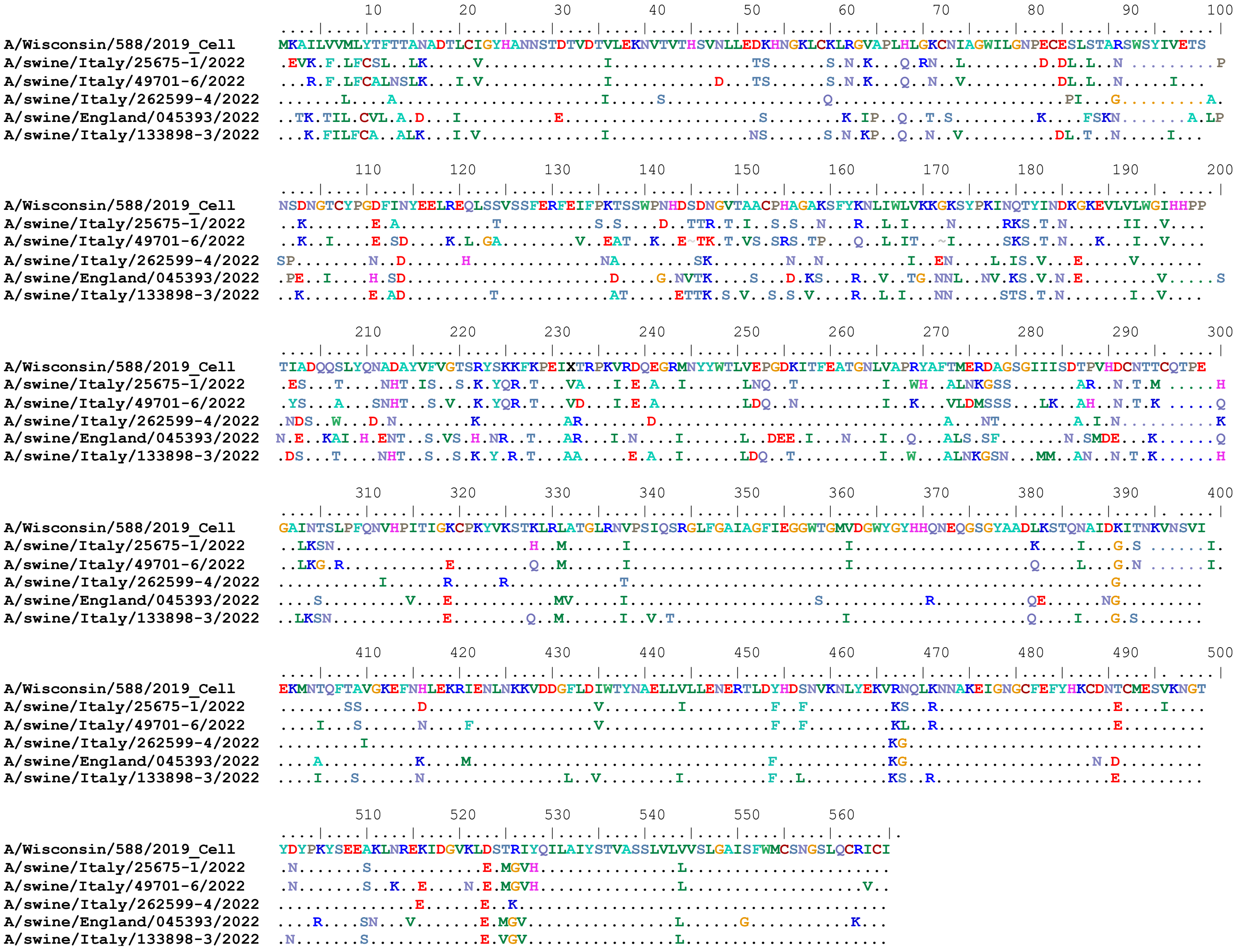

Figure S1. Alignment of HA protein sequences for the panel of SwIAV used in this study compared to A/Wisconsin/588/2019. Accession numbers for each virus can be found in Table 1.

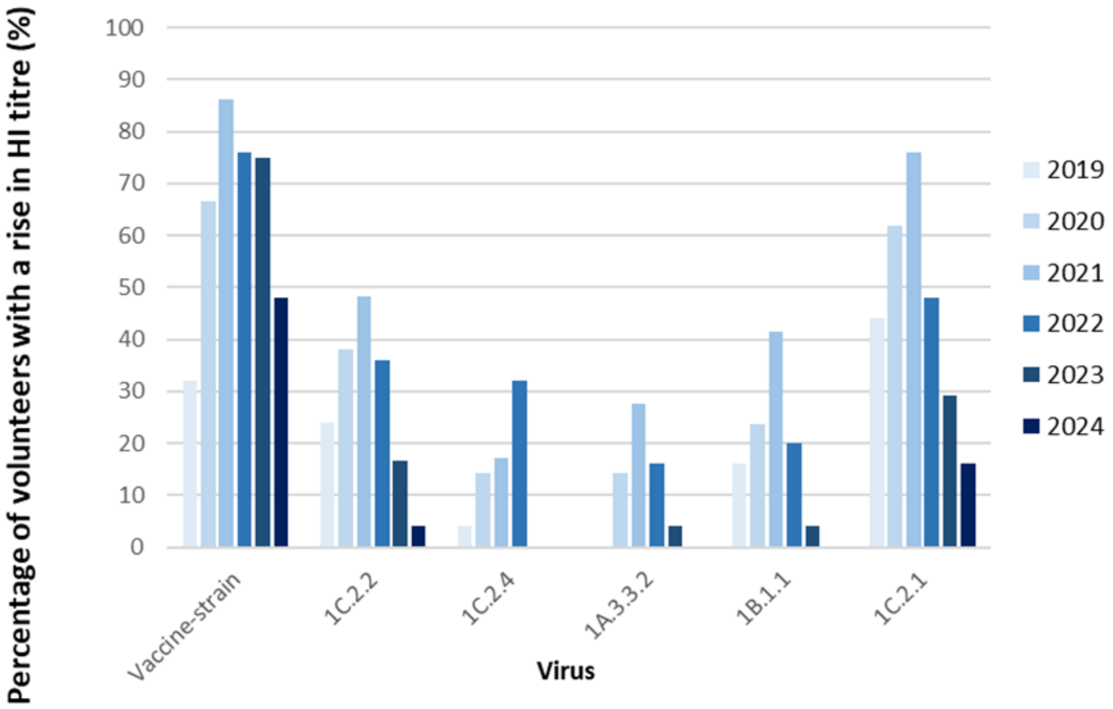

Figure S2. The percentage of donors with a rise in HI titre against the respective vaccine strain and five different clades of swine influenza A virus, following vaccination with the seasonal influenza vaccine across six years.

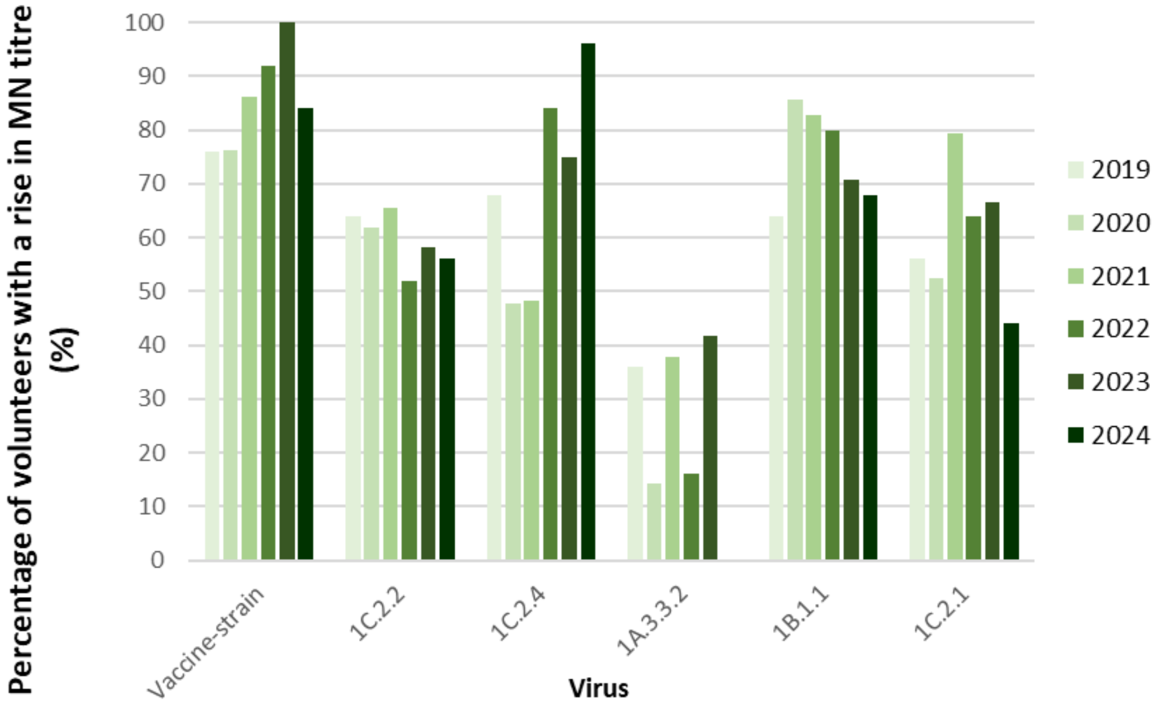

Figure S3. The percentage of donors with a rise in MN titre against the respective vaccine strain and five different clades of swine influenza A virus, following vaccination with the seasonal influenza vaccine across six years.

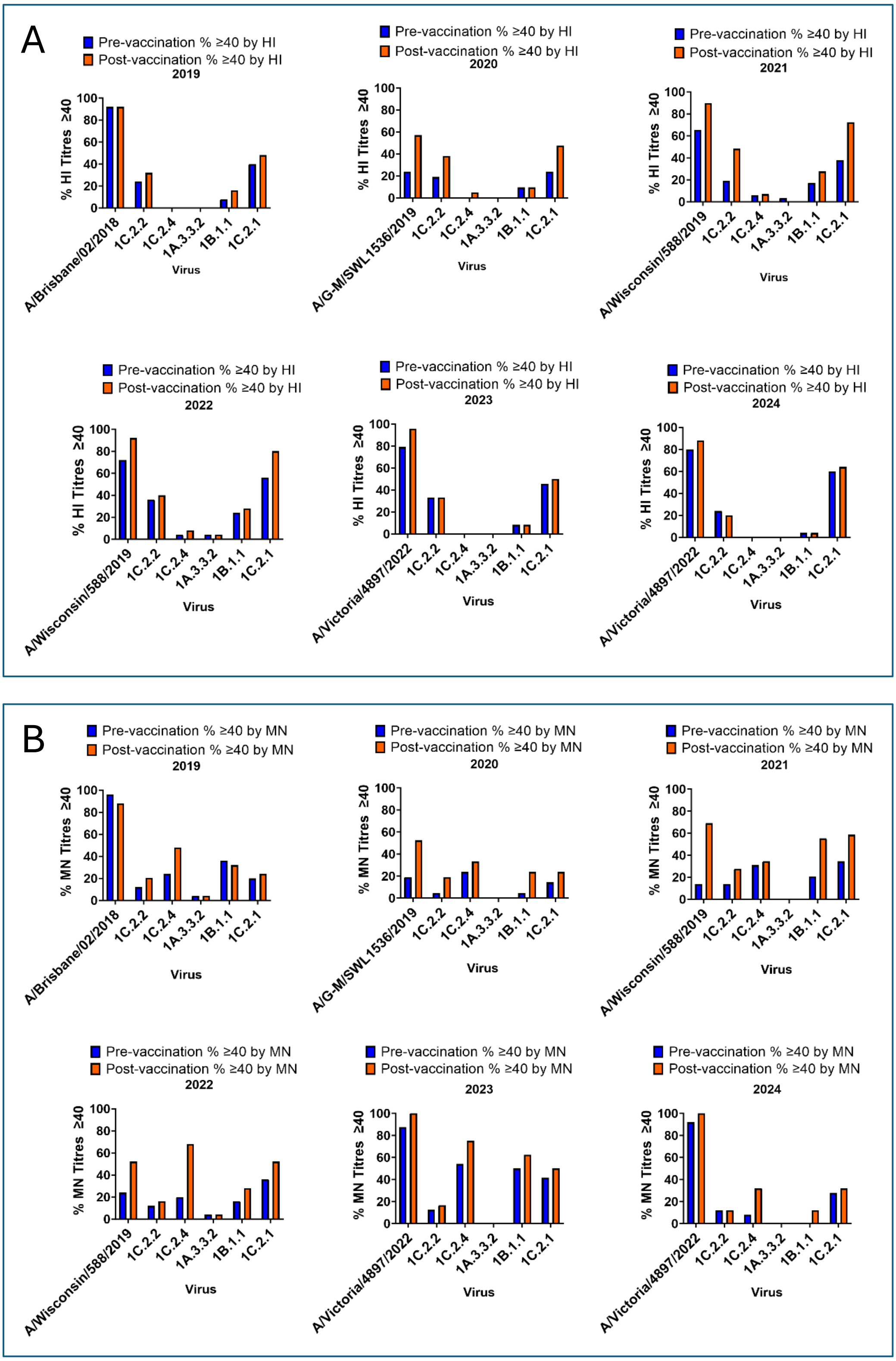

Figure S4. The percentage of HI (panel A) and MN (panel B) assay titres greater than 40 by serum panel. All five clades of swine virus and the relevant vaccine strain are included. Note, an MN titre of 40 is used as a guide and is not an established correlate of protection.

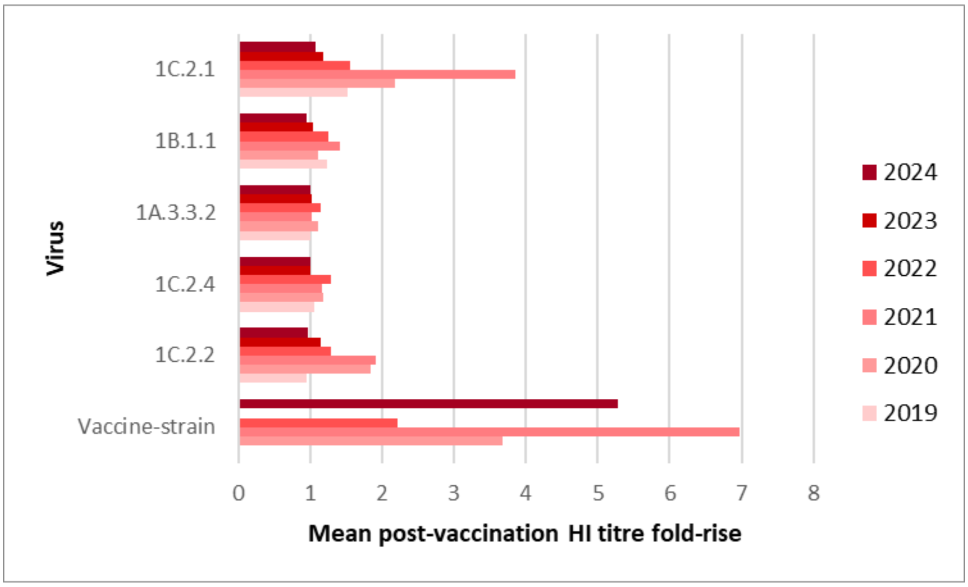

Figure S5. The mean fold-rise in HI titres for donors with pre-vaccination titres less than 40 against five different clades of swine virus and the respective vaccine strain, following vaccination with the seasonal influenza vaccine across six years. The vaccine strain is excluded where no donors had pre-vaccination MN titres less than 40.

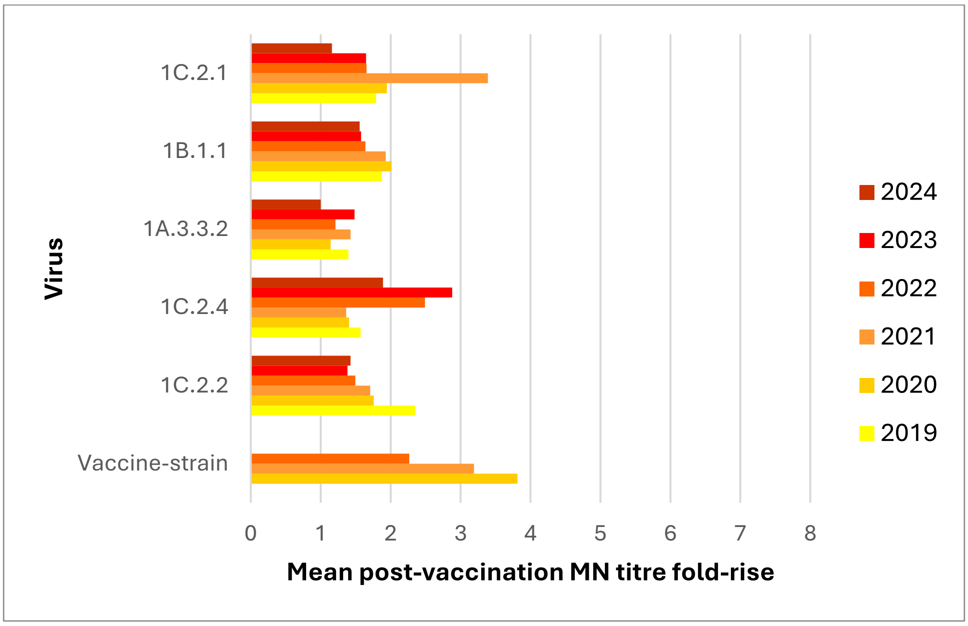

Figure S6. The mean fold-rise in MN titres for donors with pre-vaccination titres less than 40 against five different clades of swine virus and the respective vaccine strain, following vaccination with the seasonal influenza vaccine across six years. The vaccine strain is excluded where no donors had pre-vaccination MN titres less than 40.

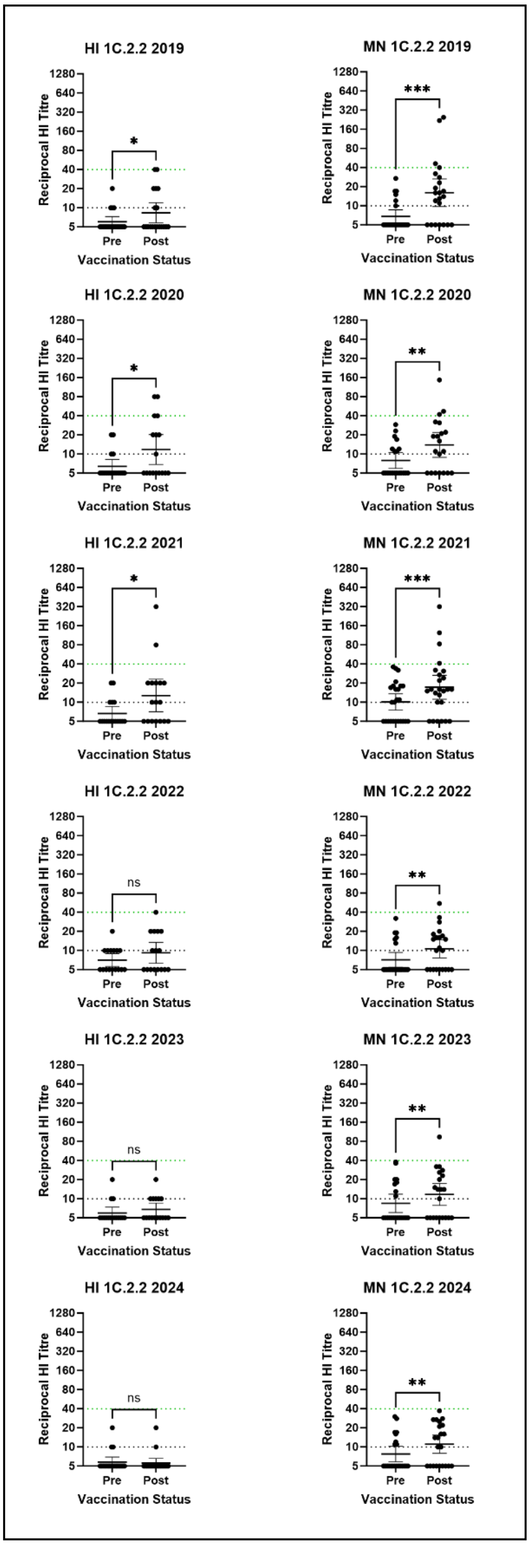

Figure S7. HI and MN assay results, using only donors with pre-vaccination titres less than 40, against the 1C.2.2 clade virus against all six human serum panels. Stars denote significance between pre- and post-vaccination titres as calculated by the Wilcoxon test: * = $p \leq 0.05$; ** = $p \leq 0.01$; *** = $p \leq 0.001$; ns = $p > 0.05$. Points represent individual titres and horizontal lines denote the GMT and 95% CI. The dotted green line represents the CoP and the dotted black line is the detection threshold.

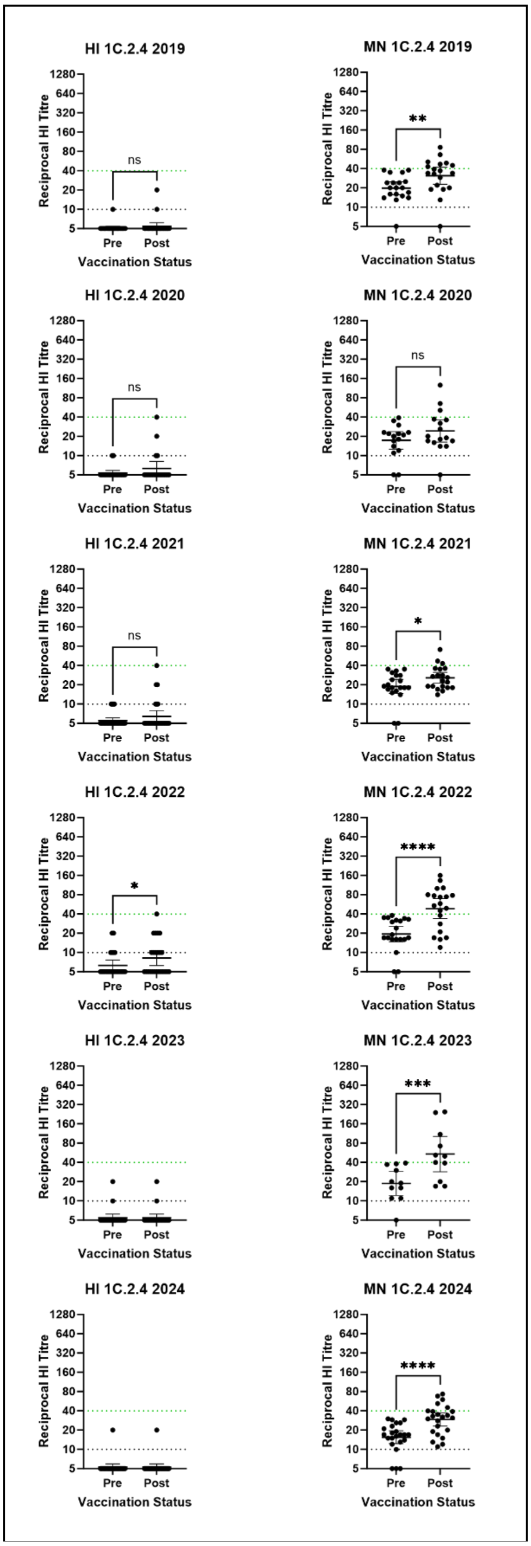

Figure S8. HI and MN assay results, using only donors with pre-vaccination titres less than 40, against the 1C.2.4 clade virus against all six human serum panels. Stars denote significance between pre- and post-vaccination titres

as calculated by the Wilcoxon test: * = p ≤ 0.05; *** = p ≤ 0.001; **** = p ≤ 0.0001; ns = p > 0.05. Points represent individual titres and horizontal lines denote the GMT and 95% CI. The dotted green line represents the CoP and the dotted black line is the detection threshold.

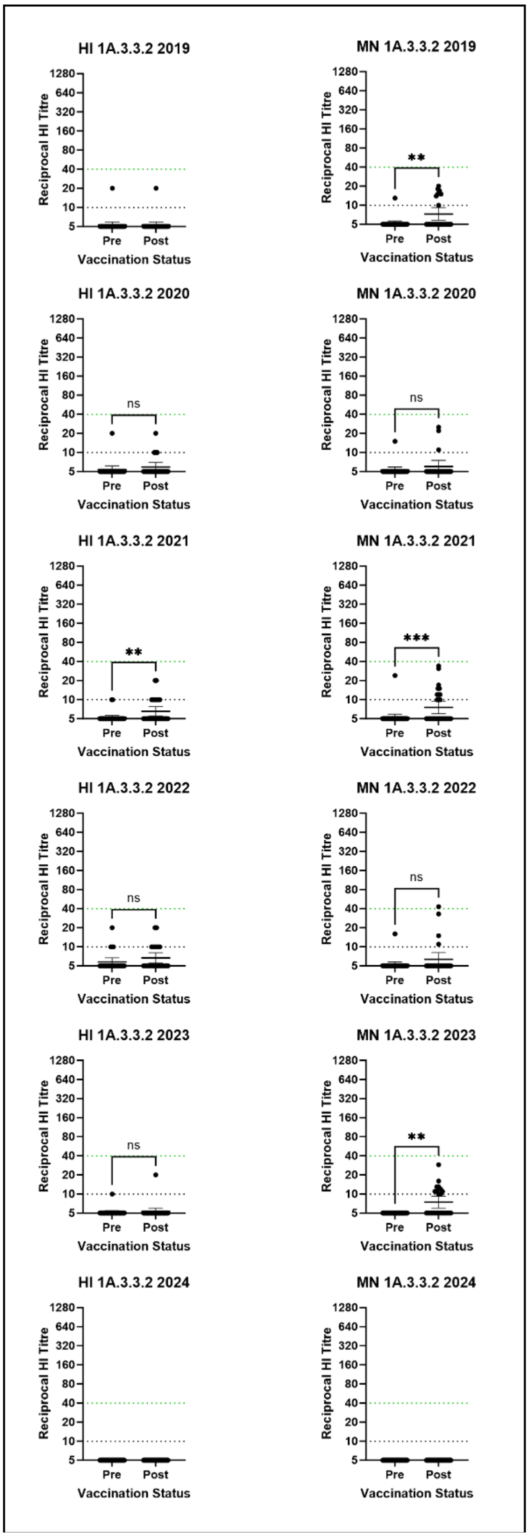

Figure S9. HI and MN assay results, using only donors with pre-vaccination titres less than 40, against the 1A.3.3.2 clade virus against all six human serum panels. Stars denote significance between pre- and post-vaccination titres as calculated by the Wilcoxon test: ** = p ≤ 0.01; *** = p ≤ 0.001; ns = p > 0.05. Points represent individual titres and

horizontal lines denote the GMT and 95% CI. The dotted green line represents the CoP and the dotted black line is the detection threshold.

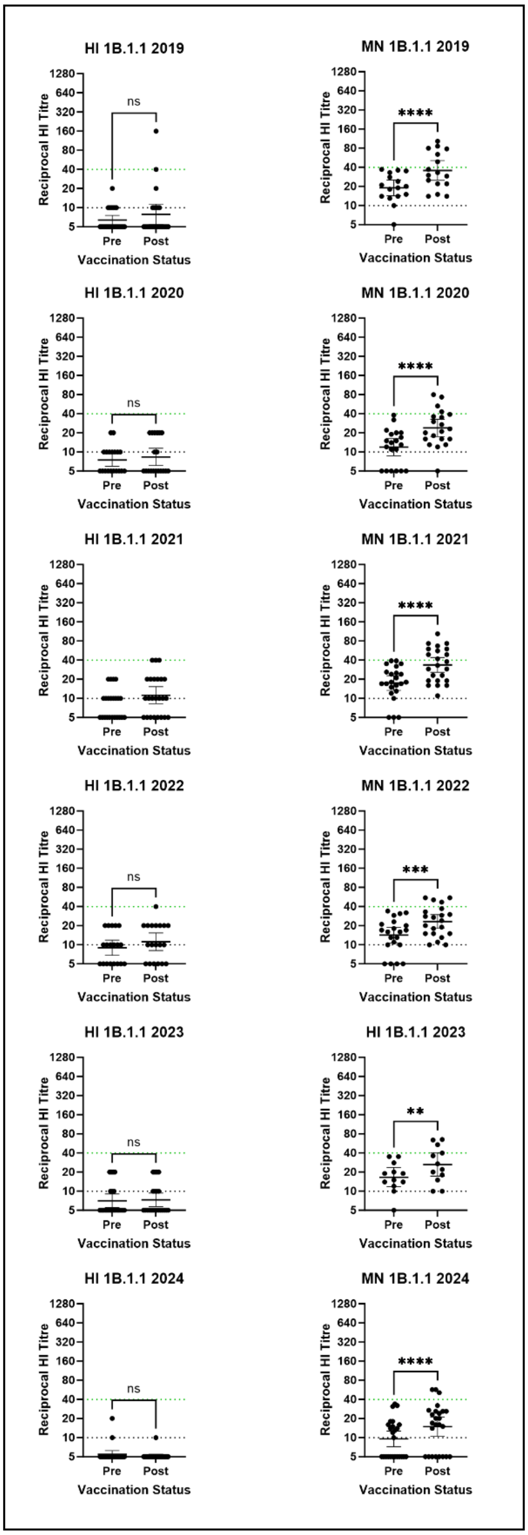

Figure S10. HI and MN assay results, using only donors with pre-vaccination titres less than 40, against the 1B.1.1 clade virus against all six human serum panels. Stars denote significance between pre- and post-vaccination titres as calculated by the Wilcoxon test: ** = p ≤ 0.01; *** = p ≤ 0.001; **** = p ≤ 0.0001; ns = p > 0.05. Points represent individual titres and

horizontal lines denote the GMT and 95% CI. The dotted green line represents the CoP and the dotted black line is the detection threshold.

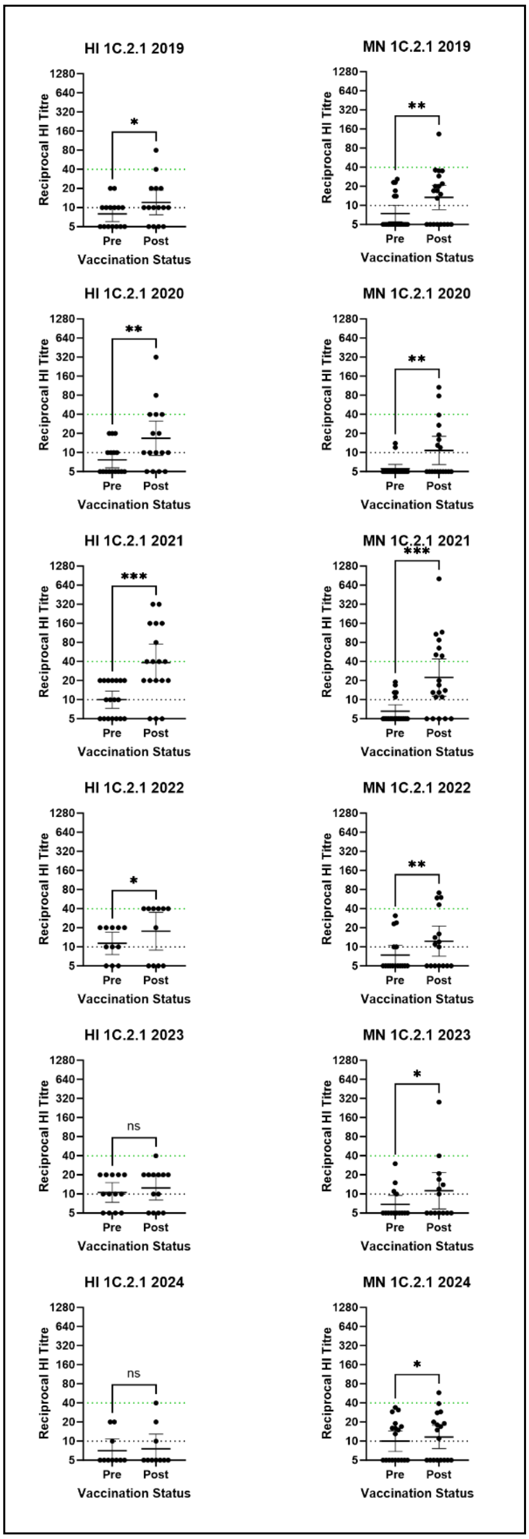

Figure S11. HI and MN assay results, using only donors with pre-vaccination titres less than 40, against the 1C.2.1 clade virus against all six human serum panels. Stars denote significance between pre- and post-vaccination titres as calculated by the Wilcoxon test: * = p ≤ 0.05; ** = p ≤ 0.01; *** = p ≤ 0.001; ns = p > 0.05. Points represent individual titres and horizontal

lines denote the GMT and 95% CI. The dotted green line represents the CoP and the dotted black line is the detection threshold.

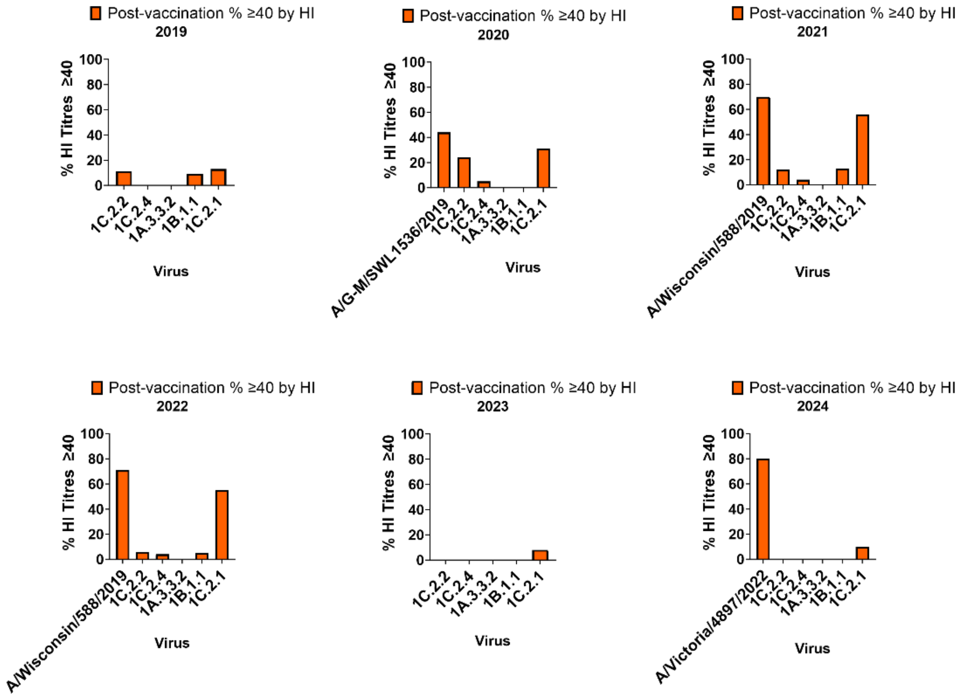

Figure S12. The percentage of post-vaccination HI assay titres greater than or equal to 40, the correlate of protection, by serum panel, adjusted to include only donors with pre-vaccination titres less than 40. All five clades of swine virus and the relevant vaccine strain are included. The vaccine strain is excluded where no donors had pre-vaccination HI titres less than 40.

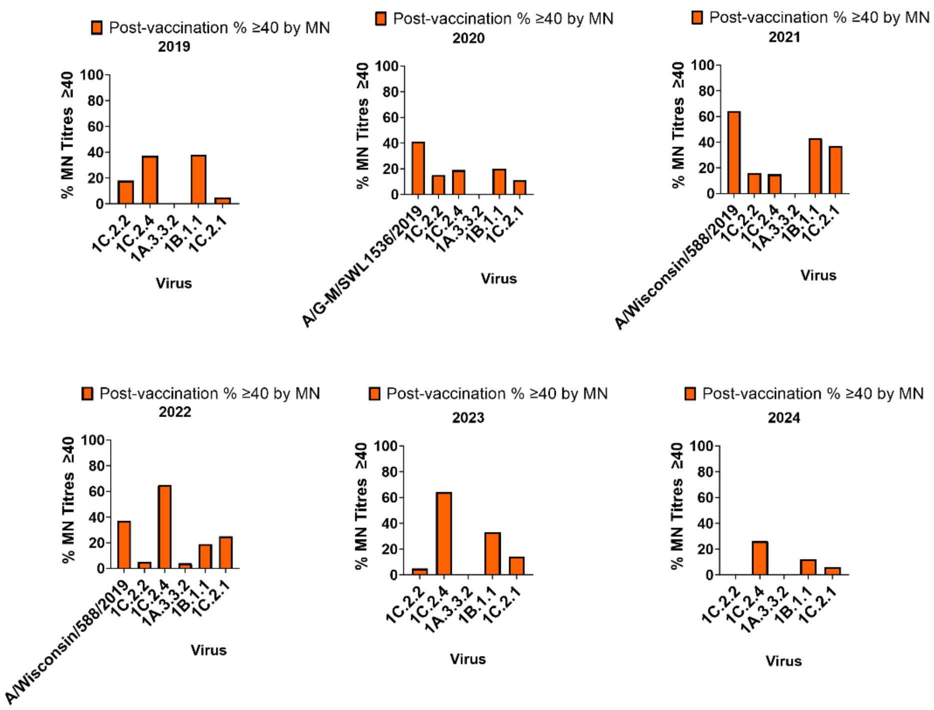

Figure S13. The percentage of post-vaccination MN assay titres greater than or equal to 40 by serum panel, adjusted to include only donors with pre-vaccination titres less than 40. All five clades of swine virus and the relevant vaccine strain are included. The vaccine strain is excluded where no donors had pre-vaccination MN titres less than 40.

Table S1. Serum panel sizes.

Serum panel year	Number of donors
2019	25
2020	21
2021	29
2022	25
2023	24
2024	25

Table S2. Serum panel sizes tested by HI when adjusted to include only donors with pre-vaccination titres less than 40 against the respective viruses.

Serum panel	Virus	Number of donors
2019	A/Brisbane/02/2018	0
	1C.2.2	19
	1C.2.4	25
	1A.3.3.2	25

	1B.1.1	23
	1C.2.1	15
2020	A/G-M/SWL1536/2019	16
	1C.2.2	17
	1C.2.4	21
	1A.3.3.2	21
	1B.1.1	19
	1C.2.1	16
2021	A/Wisconsin/588/2019	10
	1C.2.2	17
	1C.2.4	28
	1A.3.3.2	28
	1B.1.1	24
	1C.2.1	18
2022	A/Wisconsin/588/2019	7
	1C.2.2	16
	1C.2.4	24
	1A.3.3.2	24
	1B.1.1	19
	1C.2.1	11
2023	A/Victoria/4897/2022	0
	1C.2.2	16
	1C.2.4	24
	1A.3.3.2	24
	1B.1.1	22
	1C.2.1	13
2024	A/Victoria/4897/2022	5
	1C.2.2	19
	1C.2.4	25
	1A.3.3.2	25
	1B.1.1	24
	1C.2.1	10

Table S3. Serum panel sizes tested by MN when adjusted to include only donors with pre-vaccination titres less than 40 against the respective viruses.

Serum panel year	Virus	Number of donors
2019	A/Brisbane/02/2018	0
	1C.2.2	22
	1C.2.4	19
	1A.3.3.2	24
	1B.1.1	16
	1C.2.1	20
2020	A/G-M/SWL1536/2019	17
	1C.2.2	21
	1C.2.4	21
	1A.3.3.2	21
	1B.1.1	21
	1C.2.1	21
2021	A/Wisconsin/588/2019	29
	1C.2.2	25

	1C.2.4	20
	1A.3.3.2	29
	1B.1.1	23
	1C.2.1	19
2022	A/Wisconsin/588/2019	19
	1C.2.2	22
	1C.2.4	20
	1A.3.3.2	25
	1B.1.1	21
	1C.2.1	16
2023	A/Victoria/4897/2022	0
	1C.2.2	21
	1C.2.4	11
	1A.3.3.2	24
	1B.1.1	12
	1C.2.1	14
2024	A/Victoria/4897/2022	0
	1C.2.2	22
	1C.2.4	23
	1A.3.3.2	25
	1B.1.1	25
	1C.2.1	18
